# Integrating Virtual Reality Simulation, Online Learning, and Group Reflection to Strengthen Dementia Care in Nursing Homes: Mixed Methods Pilot Study

**DOI:** 10.2196/86672

**Published:** 2026-04-27

**Authors:** Li-Mei Chen, Megumi Inoue

**Affiliations:** 1 Department of Social Work, College of Public Health, George Mason University, 4400 University Drive MS1F8, Fairfax, VA, 22030, United States, 1 7039931078

**Keywords:** AD/ADRD, relationship-centered care, extended reality, empathy, workforce, long-term care, Alzheimer disease

## Abstract

**Background:**

Long-term care facilities are increasingly caring for persons living with dementia as this population grows. Frontline care workers provide most hands-on support, yet they often have limited access to formal dementia education and training. Traditional training formats frequently fail to support experiential learning or accommodate the linguistic, cultural, and demographic diversity of the long-term care workforce.

**Objective:**

This mixed methods pilot study examined the effects of the combined use of online learning, immersive virtual reality (VR) simulation, and facilitated group discussions on the training and preferred learning formats. In particular, this study tested whether training based on relationship-centered care (eg, emphasizing the importance of mutual respect, empathy, and shared humanity) in care relationships embodied in the immersive VR simulation allows staff to experience dementia-related cognitive and sensory changes from the perspective of persons living with dementia.

**Methods:**

A total of 21 certified nursing assistants from 1 US nursing home participated in a 3-month mixed methods intervention. Empathy and knowledge were measured using pre- and postintervention standardized tests; qualitative feedback was collected through open-ended surveys and group discussions.

**Results:**

Participants were predominantly female, Black certified nursing assistants with approximately 68% reporting 8 years or more of care experience. Among the 76.2% (16/21) of the participants who completed the pre- and postintervention surveys, empathy scores increased from pretest (mean 5.31, SD 0.74) to posttest (mean 5.51, SD 0.61). The mean difference of 0.20 (SD 0.43) did not reach statistical significance (*t*_15_=1.88; *P*=.08), but the effect size was moderate (Cohen *d*_z_=0.47, 95% CI −0.03 to 0.43). Dementia knowledge scores also increased from pretest (mean 5.50, SD 2.37) to posttest (mean 5.94, SD 2.11), with a mean difference of 0.44 (SD 1.63), which was not statistically significant (*t*_15_=1.07; *P*=.30), and demonstrated a small effect size (Cohen *d*_z_=0.27, 95% CI −0.43 to 1.31). Qualitative findings revealed that participants perceived the VR training as engaging and emotionally impactful. Participants described reframing their understanding of dementia, reporting reduced stigma and increased empathy toward persons living with dementia. Many noted that experiencing dementia-related symptoms through VR helped them better understand residents’ behaviors and respond with greater compassion. Participants expressed a strong preference for immersive VR and facilitated group discussions over online modules, and cultural differences in the VR scenarios were not perceived as barriers to learning.

**Conclusions:**

While preliminary, these findings suggest that combining relationship-centered care with immersive VR may enhance empathy and engagement among staff, particularly when paired with facilitated discussion and plain language explanations. This multimodal model appears particularly valuable for supporting empathic learning within diverse and experienced workforces. Larger, multisite studies with sustained follow-up are needed to determine long-term effects and optimize training for linguistically and culturally diverse workforces.

## Introduction

Dementia is one of the most pressing global public health issues, affecting an estimated 55.2 million people worldwide [1]. Impaired cognitive functioning is one of the major contributing factors leading to nursing home placement among older adults worldwide [2], and the prevalence rate of persons with cognitive impairment in nursing homes was 21.2% in 2022 [3]. In the United States alone, this translates to approximately 3 million persons living with dementia currently residing in nursing homes, a number expected to increase with population aging and rise in dementia prevalence [4].

The quality of care is contingent upon the direct care staff. This was underscored by the Biden administration’s passage of a law in 2022 to improve nursing home care by increasing staffing levels [5], although the policy was repealed under the Trump administration in 2025 [6]. While the direct relationship between staffing levels and a nursing home’s quality of care is under-studied [7], existing evidence constantly demonstrates that nursing staff are critical to the delivery of high-quality care [8]. Specifically with care for persons living with dementia, the skills and attitudes of direct care staff are central to maintaining their quality of life [9,10]. Recent work by Mukamel et al [4] further suggests that nursing homes with specialized care in dementia provide better care for their residents living with dementia, highlighting that targeted resources and staffing focused on dementia care are effective.

Despite their central role, the vast majority of nursing home staff work under challenging conditions in which they navigate communication barriers, behavioral and psychological symptoms of dementia (BPSD), and the emotional demands of providing care [11]. These stressors contribute to staff stress and burnout [11-15] and are among the leading causes of high turnover rates in nursing homes [16,17]. Workforce instability, in turn, undermines continuity of care and limits opportunities for skill training and development.

A recent study on staff in dementia care facilities reported that in-service training focusing on capacity building has been shown to improve staff attitudes, knowledge, and competence while potentially mitigating job stress [18]. Evidence on BPSD-specific training has demonstrated the value of structured, skill-based approaches that can be integrated into demanding work routines [19]. However, high-quality dementia care extends beyond task competence. It requires the ability to connect, communicate, and build relationships grounded in empathy and trust [20,21]. Traditional dementia training, whether it is delivered in classrooms, via printed materials, or through online modules, often falls short in fostering such relational and empathetic skills [22]. Moreover, existing trainings have often been found to have limited impact on relational skills and sustained behavior change [23,24]. Other common limitations of traditional training include a lack of immersive, experiential learning; minimal adaptation to linguistic and cultural diversity; and insufficient reinforcement for long-term knowledge retention [24-27].

In response to the need for more empathic approaches to care, the health care field has increasingly adopted relationship-centered care. This approach grew out of a research program and agenda to encourage the development or expansion of educational programs that reflect an integrated biomedical-psychosocial perspective—an integrated approach to patient care [28]. Research has shown that empathy among medical students and residents often declines over time as they progress through medical training, particularly in the context of time pressure, heavy workloads, and emotional fatigue [29]. Relationship-centered care was introduced as a corrective framework that builds on understanding the centrality of relationships, recognizing that both patients and physicians in the care relationship matter. In dementia care, relationship-centered care emphasizes the importance of building long-lasting strong, trusting relationships between persons living with dementia and care workers. These personal connections allow residents to feel more valued and understood, which can significantly enhance their emotional well-being and overall satisfaction with care [30].

Relationship-centered care is conceptually distinct from the well-known concept of person-centered care because person-centered care does not always emphasize the importance of human connection and personhood in relationships [29]. Training in relationship-centered care positions empathy, trust, and nuanced moment-to-moment communication as central to dementia care training, such as through eye contact, appropriate touch, and adaptive speech. Training in empathy and empathetic communication has been shown to significantly improve participant empathy, attitudes toward patients with dementia, and certain verbal and nonverbal communication skills [31]. There is also strong evidence that empathetic communication training led staff to have enhanced positive behavior and satisfying interactions with persons living with dementia [32].

Furthermore, there is growing evidence suggesting that multimodal training aligned with relationship-centered care principles can enhance care processes, suggesting that experiential and skill-focused education is an important complement to didactic content [33]. In particular, training with in-person feedback and guided reflection have enhanced the attained skills [32]. Debriefing sessions in simulated training for health care professionals have proved effective in both technical and nontechnical skills [34]. Guided group discussions after training for medical trainees have been found to form professional identity and create professional socialization [35].

Simulation-based education is widely recognized for improving communication and empathy in health care [36]. Virtual reality (VR), in particular, allows learners to “step into the shoes” of persons living with dementia and experience their cognitive, sensory, and emotional challenges. Research suggests that VR can enhance empathy, dementia knowledge, and reflective practice [37,38], although its effectiveness may vary by staff characteristics such as age and primary language [39]. Despite its promise, most VR interventions have been implemented with students or informal caregivers, not with nursing home direct care staff, who provide most hands-on dementia care. Barriers such as cost, equipment needs, and staffing constraints have limited access to such training in long-term care settings [40,41].

Recent advances have strengthened the feasibility of VR simulation-based learning, which immerses participants in the lived experience of persons living with dementia. A dementia-focused review by Huang et al [37] found that VR can improve empathy, knowledge, and reflective practice, although challenges such as simulation sickness and equipment comfort must be addressed in nursing home contexts. A broader meta-analysis in health care education indicates that VR can outperform non-VR methods in improving knowledge, skills, and learner satisfaction [38]. Microlearning formats, which are short, focused VR modules, have been linked to higher engagement and retention [42]. Similarly, just-in-time simulation supports targeted learning refreshers close to the point of care [43], aligning with this study’s monthly VR-plus-discussion structure.

Despite growing interest in the use of VR in dementia care training, several critical gaps exist in the literature. First, prior VR-based dementia training studies have mostly focused on students, family caregivers, or health care professionals outside of long-term care, with limited attention given to direct care staff [37]. This omission is clearly consequential, as direct care staff in nursing homes are the ones who deliver most hands-on support to residents living with dementia. By focusing on this workforce, this study addresses a critical gap in the literature and responds to a population with unique training needs and operational constraints. Moreover, their training opportunities are restricted by time, staffing shortages, deprioritization of dementia education, and chronic underfunding for direct care staff, underscoring the need for empirically tested, accessible, and practice-relevant interventions that meet the needs of this workforce.

Second, few prior interventions have taken a fully integrated approach to learning. There are studies that have offered online education or stand-alone VR experiences, but few studies have examined the effects of VR when embedded into comprehensive training that integrates cognitive learning with simulated experience and reflective processing [42,43]. The absence of such a learning model limits the understanding of how VR can impact direct care workers’ approach to care for persons living with dementia. Specifically, there is a lack of data on how VR effects changes in knowledge acquisition on dementia care, promotes empathy, and encourages translation of relationship-centered care skills into daily practice.

Third, while empathy is often discussed as a key outcome of dementia training, it is not consistently measured alongside factual knowledge gains. Many studies assess empathy or attitudes in isolation, making it difficult to determine whether VR-based training simultaneously strengthens affective capacities (eg, empathic perspective taking) and cognitive competencies (eg, understanding effective communication approaches for persons living with dementia). This lack of dual-outcome assessment constrains conclusions about the comprehensive educational value of VR interventions [24,27,37,38].

To address these gaps, this study aimed to evaluate a multimodal dementia care training that integrated relationship-centered care–based online modules, immersive VR simulations, and facilitated group reflection specifically targeting direct care workers in nursing homes. This study tested whether VR-enhanced training produced improvements in primary outcomes—empathy and communication knowledge. This design allows for hypothesis-driven examination of VR’s potential role as a mechanism for increasing empathic understanding and strengthening dementia-specific communication knowledge among direct care staff working with persons living with dementia. The paucity of reporting guidelines on VR led us to use the reporting guidelines by Vlake et al [44] on early clinical evaluation on the use of extended reality, which encompasses VR, mixed reality, and augmented reality, to report our study (Multimedia Appendix 1).

## Methods

### Overview

This mixed methods pilot study used a pretest-posttest design to evaluate a 3-month dementia training program for nursing home direct care staff. The quantitative component measured changes in empathy and dementia communication knowledge before and after the intervention. The qualitative component consisted of focus group interviews conducted at posttest to explore participants’ perceptions of the program’s content, delivery, and relevance to their work.

### Recruitment

The study was conducted in a nursing home located in northern Virginia. Conducting studies in nursing homes, especially the entry phase of recruitment, has been documented as difficult due to organizational, administrative, and staff barriers [45]. Specifically, clear support from top management, ability to coordinate shifts with colleagues, and maneuvering high workload and turnover rates have been reported as important conditions for successful research [46]. Hence, this study used a nonprobability, convenience sampling approach. Recruitment was conducted through public posting and distribution of flyers that included the study description and inclusion criteria. Eligible participants were staff who (1) worked in direct care roles, (2) could communicate in English, and (3) were willing to complete all components of the training and data collection. There were no formal screening procedures used. With permission from the nursing home administrative team, flyers created by the investigators were distributed to the nursing home’s direct care staff. Interested staff members volunteered to participate in the study. Administrative staff also helped with recruitment by speaking directly to care staff and encouraging participation. Recruitment was coordinated through facility administrators. In particular, developing a trusting personal contact with one of the key administrators was very helpful to manage the recruitment process [45].

### Intervention

The training program was conducted by the principal investigator and coinvestigator once a month over the course of 3 months from March to May 2025 using a combination of the 3 complementary learning formats described below. This multimodal approach was designed to layer conceptual understanding (online module), experiential insight (VR), and application (group discussion).

#### Online Module (Monthly)

Three online modules were created by the investigators using Microsoft PowerPoint slides and narrated and recorded using Zoom (Zoom Video Communications). These videos were then uploaded to the YouTube channel. They were made available through a handout with QR codes linked to the YouTube videos. Self-paced content covered relationship-centered care principles, effective communication strategies (eg, posture, eye contact, touch, and adapted speech), and approaches to BPSD management.

#### VR Simulation (Monthly)

The simulation was delivered during in-person sessions using Jolly Good Inc’s dementia care VR platform [47]. Our VR content focused on the use of real actors, whereas many of the VR simulation trainings in dementia use avatars. Past research findings suggest that older adults are less likely to connect with avatars, thus making their VR experience less effective [48,49]. The VR videos were recorded in Japan using Japanese actors. Three scenarios placed participants in the perspective of a person living with dementia. The first scenario simulated visual hallucinations, a symptom observed in persons with moderate to severe Lewy body dementia. The second simulated paranoia and object misrecognition, symptoms observed in persons with moderate to severe dementia. In the final scenario, the VR video alternated between 2 perspectives: an aide providing care and a resident living with dementia who was experiencing confusion. It highlighted how the same interaction can feel from each viewpoint.

#### Facilitated 60-Minute Group Discussion (Monthly)

Immediately following each VR session, the principal investigator guided debriefing on the VR experience, checked for cybersickness, encouraged peer sharing of strategies, and linked learning to everyday practice.

### Measures

Pre- and posttest outcomes were measured using 2 standardized tests. Empathy was assessed using the 10-item Jefferson Scale of Empathy–Healthcare Provider Version [50], which uses a 7-point Likert scale (1=“strongly disagree”; 7=“strongly agree”). Items reflecting a lack of empathy were reverse scored. Higher scores indicated greater empathy. Dementia communication knowledge [51] was measured using a 10-item multiple-choice test focusing on effective communication techniques with persons living with dementia. Scores reflected the number of correct responses. We also collected participants’ demographics, including age, gender, race and ethnicity, language spoken at home, years of experience in direct care, work status, and personal experiences with persons living with dementia. Additionally, we conducted focus group interviews with open-ended questions exploring learning experiences, perceived applicability, and perceived changes in attitudes or behaviors. Three open-ended questions were posed: (1) “How was the overall training?” (2) “What did you think about the VR experience?” (3) “What did you think about the online videos?”

### Procedures

Baseline surveys were conducted in February 2025. Training sessions took place monthly from March 2025 to May 2025, with each month’s in-person session including VR and discussion. Postintervention surveys and focus groups were completed in May 2025. These sessions were all conducted before or after the participants’ work shifts. Jolly Good Inc provided the VR headsets, software, and on-site technical support.

### Ethical Considerations

The study protocol was determined to be exempt from in George Mason University’s Institutional Review Board (STUDY00000298). However, we collected informed consent from our participants, which delineated their rights to terminate participation at any time and to be protected in terms of privacy and confidentiality. Participation was voluntary, and all data were deidentified prior to analysis. All participants received a US $100 addition to their salaries at the start of the training and a US $100 gift card upon completion of the training.

### Analysis

Quantitative analyses were performed in the SPSS statistical software (IBM Corp). Paired-sample 2-tailed *t* tests were conducted to compare pre- and postintervention empathy and knowledge scores for participants who completed both assessments. Effect sizes were calculated using the Cohen *d*.

After the intervention, qualitative focus group interviews were conducted, videotaped, transcribed verbatim, and analyzed using thematic analysis to identify patterns related to perceived learning, feasibility, and practical application. The initial analysis was conducted by the principal investigator, and the results were then discussed and validated by the team of investigators through team discussion meetings. Data from both strands of the study were integrated during interpretation to provide a comprehensive understanding of program outcomes.

## Results

### Descriptive Results: Demographics

A detailed demographic profile of the participants is provided in Table 1. The study included 21 participants, with 76.2% (16/21) completing both pre- and postassessments. Participants’ mean age was 45.8 (SD 15.0) years for the full sample and 45.7 (SD 16.3) years for the complete-case subset. Most participants were female (19/21, 90.5% in the full sample; 15/16, 93.8% in the complete-case subset) and identified as Black or African American (20/21, 95.2% in the full sample; 15/16, 93.8% in the complete-case subset). Nearly half (10/21, 47.6%) of the full sample reported more than 16 years of experience in dementia care, with a similar distribution in the matched subset (7/16, 43.8%). Informal or on-the-job dementia training was the most common form of prior training (15/21, 71.4% in the full sample; 11/16, 68.8% in the complete-case subset). Primary work shifts were split between day (8/21, 38.1%) and evening (13/21, 61.9%) in the full sample, whereas the complete-case subset predominantly worked day shifts (11/16, 68.8%). Regarding the number of residents living with dementia in their care, the full sample reported that 28.6% (6/21) cared for 1 to 3 residents, 14.3% (3/21) cared for 4 to 6 residents, 52.4% (11/21) cared for 7 to 10 residents, and 4.8% (1/21) cared for more than 10 residents. The complete-case subset had a similar distribution. Most participants (18/21, 85.7% in the full sample; 14/16, 87.5% in the complete-case subset) spoke English at home, but it was an African dialect, not American English. Over one-third (7/21, 33.3% in the full sample; 5/16, 31.3% in the complete-case subset) reported a personal connection to dementia.

**Table 1.** Demographic profile of the participants who responded to the preintervention survey (entire sample) and both the pre- and postintervention surveys (paired sample=37).

Characteristic	Entire sample (n=21)	Paired sample (n=16)
Age (years), mean (SD)	45.8 (14.98)	45.67 (16.33)
Gender, n (%)		
Women	19 (90.5)	15 (93.8)
Men	2 (9.5)	1 (6.3)
Race, n (%)		
Black or African American	20 (95.2)	15 (93.8)
Hispanic or Latinx	1 (4.8)	1 (6.3)
Experience (years), n (%)		
1-3	3 (14.3)	3 (18.8)
4-7	2 (9.5)	2 (12.5)
8-15	6 (28.6)	4 (25.0)
≥16	10 (47.6)	7 (43.8)
Previous dementia training, n (%)		
Formal	3 (14.3)	2 (12.5)
Informal	15 (71.4)	11 (68.8)
None	3 (14.3)	3 (18.8)
Primary shift, n (%)		
Day	8 (38.1)	11 (68.8)
Evening	13 (61.9)	5 (31.3)
Residents with dementia in their care per shift, n (%)		
1-3	6 (28.6)	5 (31.3)
4-6	3 (14.3)	2 (12.5)
7-10	11 (52.4)	8 (50.0)
>10	1 (4.8)	1 (6.3)
Primary language spoken at home, n (%)		
English	18 (85.7)	14 (87.5)
Spanish	1 (4.8)	1 (6.3)
Other	2 (9.5)	1 (6.3)
Personal connection to dementia, n (%)		
Yes	7 (33.3)	5 (31.3)
No	14 (66.7)	11 (68.8)

### Bivariate Results

Table 2 reports the standardized test scores. For the paired-sample *t* tests, 16 participants who completed both the pre- and postassessments were included. Empathy was assessed using a 10-item composite scale, including 4 reverse-scored items with higher values indicating greater empathy. The results showed a slight increase in empathy from pretest (mean 5.31, SD 0.74) to posttest (mean 5.51, SD 0.61). The mean difference was 0.20 (SD 0.43) points, with a 95% CI for the mean difference of −0.03 to 0.43. This change did not reach statistical significance (*t*_15_=1.88; *P*=.08), although the corresponding effect size was in the small to moderate range (Cohen *d*=0.47).

**Table 2.** Empathy and dementia knowledge test results of the paired sample of participants (n=16)^a^.

Measure	Before, mean (SD)	After, mean (SD)	*t* test (*df*)	*P* value	Cohen *d*
Empathy (1-7)	5.31 (0.74)	5.51 (0.61)	1.88 (15)	.08	0.47
Knowledge (1-10)	5.50 (2.37)	5.94 (2.11)	1.07 (15)	.30	0.27

a *P* values are 2 tailed. Cohen *d* values for paired-sample *t *tests are calculated using the SD of the pretest scores. “Paired” refers to participants who completed both the pre- and posttests.

Dementia knowledge was assessed using 10 items, with correct responses scored as 1 and incorrect responses scored as 0, yielding a total score ranging from 0 to 10. Knowledge scores increased from pretest (mean 5.50, SD 2.37) to posttest (mean 5.94, SD 2.11). The mean difference of 0.44 (SD 1.63) points, with a 95% CI of −0.43 to 1.31, was not statistically significant (*t*_15_=1.07; *P*=.30). The associated effect size was small (Cohen *d*=0.27).

### Qualitative Results

Thematic analysis identified three main themes: (1) reframing dementia, (2) growing empathy and understanding, and (3) preferred training formats.

#### Reframing Dementia

Participants described a shift in how they perceived dementia, moving away from viewing it as a problem or a source of shame toward a more compassionate perspective. For some, this shift involved recognizing that dementia is not a personal failing but a condition requiring understanding:

This training let me see a more positive and compassionate attitude towards persons with dementia. It reduced the stigma [of dementia]...like something to look down upon. I have more empathy [after the training].

Others noted that the training challenged their assumptions and helped normalize dementia as part of aging rather than something to be feared or hidden.

#### Growing Empathy and Understanding

The VR experience prompted participants to think differently about their residents, fostering a deeper sense of empathy. Some reflected on how the training helped them better understand the behaviors of persons living with dementia:

I always wanted persons with dementia to be a little bit nice to me. But now I know why they are who they are.... I think about them more.

One participant summed up their overall experience:

We learned a lot from it. I see everything good, I understand everything good.

#### Preferred Training Formats

Although most participants admitted that they did not watch the online modules as requested, they expressed a clear preference for training that included immersive VR and facilitated group discussions. They emphasized that the in-person, experiential format was more engaging and memorable than the online content alone.

Interestingly, despite the VR videos being dramatized in Japanese settings, participants reported that the cultural context did not limit their learning. The symptoms and scenarios depicted were perceived as universally relevant:

I see the same symptoms and problems.

## Discussion

### Principal Findings

This pilot study explored the effect of the integration of relationship-centered care training with immersive learning on direct care staff’s empathy and dementia knowledge. The study participants were predominantly female, Black care workers with over 16 years of experience in the field. Many learned about dementia primarily through informal, on-the-job training. They cared for multiple persons living with dementia per shift, reflecting the high emotional and physical demands typical of their roles.

As a pilot study, this investigation was designed to assess feasibility and generate preliminary estimates of intervention effects rather than test definitive hypotheses. Accordingly, the sample size (n=16) provided sufficient power to detect only large within-person effects, and the absence of statistically significant changes in empathy and dementia knowledge outcomes was anticipated. Therefore, emphasis is placed on effect size estimation and CIs to inform the design of a future adequately powered study.

While the overall changes in empathy and knowledge did not reach statistical significance, both moved in the expected positive direction. The small to moderate effect size for empathy (Cohen *d*=0.47) suggests that there may be meaningful benefits even within the short time frame of this intervention. This aligns with findings from relationship-centered care programs that foster personhood and potentially boost self-efficacy [52,53].

The qualitative findings complement the quantitative results by providing context for the small but positive changes observed in empathy and knowledge scores. Participants described the VR training as reframing their perceptions of dementia; reducing stigma; and prompting more compassionate, person-centered perspectives. Many favored interactive, immersive formats over online modules. Emotional learning appeared to be a particular strength of the VR component even though the scenarios were set in an Asian cultural context.

The knowledge effect (Cohen *d*=0.27) was more modest, which mirrored the observation by Stafford et al [54] that VR can be engaging but not always more effective than traditional dementia training. Given that most participants spoke African dialects of English and had limited baseline knowledge of dementia, traditional mainstream, web-based training methods or handouts and content written or explained in formal English may be less effective. A combination of visual learning using VR and more time spent on explaining dementia knowledge in simple English during group sessions may improve comprehension and retention.

The modest improvement observed in dementia communication knowledge (Cohen *d*=0.27) should also be interpreted in light of the qualitative findings. Several participants reported that they did not complete the online modules as intended and, instead, relied primarily on the in-person VR and facilitated discussion sessions. Therefore, limited engagement with the asynchronous modules may partially explain the small knowledge effect size observed in the quantitative analysis. This finding highlights an important structural challenge in dementia workforce training. Although online modules are often used to increase accessibility, direct care staff in long-term care settings often have limited time during work hours to access and complete them as intended. Participants indicated that training was most feasible when administrators allowed them to attend sessions during paid work time. Taken together, these mixed methods findings suggest that while immersive VR and facilitated discussion may promote emotional learning and engagement, knowledge acquisition through online modules may require stronger organizational support and protected training time to ensure consistent participation and successful attainment of training objectives.

The findings also speak about broader workforce development needs. This was an experienced, committed workforce, yet access to formal training opportunities remained limited. For certified nurse assistants, training was often feasible if administrators freed them from floor duties during work hours. Appropriate time and space for structured, evidence-based education can strengthen both empathy and knowledge outcomes. Past literature also points to stronger benefits when coupled with sustained practice and reinforcement [55]. Future programs might consider extending the duration, incorporating booster sessions, and embedding other scenarios.

### Limitations

There are several study limitations. First, the training was implemented in 1 setting with a fairly homogenous workforce, which limits generalizability. Second, due to the small sample size, statistical power was limited. Participation may have been hindered by the nursing home’s initial decision not to offer direct incentives; recruitment improved once gift cards were introduced. More visible and systemic incentives should be adopted to encourage participation in training for direct care staff. Third, given that many participants began the training with relatively high empathy scores, the potential for a ceiling effect should be considered. The smaller gains in dementia knowledge may be due to the variability in baseline knowledge and the need for repeated reinforcement to consolidate new information. Nevertheless, the positive direction of change and the strong engagement from participants point to the promise of this approach.

### Conclusions

Although this was a small pilot study, the combination of immersive VR and relationship-centered care content shows potential as part of a larger, sustained training strategy. Future work should address VR accessibility and affordability for direct care staff training for those who will benefit the most. Larger studies with more diverse samples and longer follow-up periods are needed to see whether these early gains translate into lasting changes in attitudes, communication, and care practices.

## Supplementary material

10.2196/86672 Multimedia Appendix 1 RATE-XR (Reporting for the Early-Phase Clinical Evaluation of Applications Using Extended Reality) checklist.

## Abbreviations

BPSD: behavioral and psychological symptoms of dementia
VR: virtual reality
