# Supplementary material for: Integrating Virtual Reality Simulation, Online Learning, and Group Reflection to Strengthen Dementia Care in Nursing Homes: Mixed Methods Pilot Study
Source: JMIR Form Res. 2026 Apr 27;10:e86672. doi: 10.2196/86672 (PMC13118136; doi:10.2196/86672)
Supplement: Multimedia Appendix 1 [file formative-v10-e86672-s001.docx]

Appendix

Table S1. RATE-XR (reporting for the early-phase clinical evaluation of applications using extended reality) checklist.

| **Theme** | **Item No** | **Recommendation** | **Check if completed** |
| --- | --- | --- | --- |
| Title and abstract |  |  |  |
| Title | 1 | Identify the study as an early clinical evaluation, or a similar term, of an application using XRb, or a more specific term, in the title, including its intended aim. | ✓ |
| Abstract | I | Provide a (structured) summary of the study.  Consider including the following:  A concise description of the clinical problem or knowledge gap and the rationale for using an application using XR  A concise description of the study methods, including a short description of the application including its name, study population, study setting, main outcomes, and assessment methods.  A concise description of the results, including safety and harm outcomes  A short conclusion  If applicable, details about the registration of the study in a publicly available database. | ✓ |
| Introduction |  |  |  |
| Clinical problem and existing evidence | 2 | Introduce the clinical problem for which the application using XR was used, including its relevance and a description of (the efficacy of) evidence-based or commonly used interventions or the treatment as usual, which is intended to be replaced by the application using XR. | ✓ |
| Introduction of the application | 3 | Introduce the application using XR, including the following:  Hypotheses for the potential effect; how the application is expected to contribute to the clinical problem.  If available, a concise description of, or a reference to, previous research on the same (or a similar) application. | ✓ |
| Objectives | II | Specify the study objectives or hypotheses. | ✓ |
| Methods and analysis |  |  |  |
| Trial design and reporting | III | Provide a reference to ethical approval and, if available, to any (published) study protocol and registration of the study in a publicly available repository. | ✓ |
| Trial design and reporting | IV | Describe, and mention the rationale for, the study design. For clarification, it is recommended to use a flow diagram. | ✓ |
| Participants and setting | 4 | Describe the setting and locations, including country, where data were collected and processed, and where the application using XR was applied and evaluated. | ✓ |
| Participants and setting | 5a | Describe how participants were selected and recruited and provide eligibility criteria. | ✓ |
| Participants and setting | 5b | Describe who will be applying the application and whether they were trained. | ✓ |
| Intervention and procedures | 6 | Provide a description of the application, including its content, hardware, protocol, and set-up, or provide a reference to previous publications where this information is described. Consider supplementing the description with an image, figure, or film. | ✓ |
| Intervention and procedures | 7 | Describe, or provide a reference to, the development process of the application. | ✓ |
| Intervention and procedures | 8 | Describe the participant timeline in sufficient detail to allow replication, including all procedures, co-interventions (if applicable), and (follow-up) assessments. | ✓ |
| Intervention and procedures | V | Describe and give a rationale for the control conditions or provide a rationale for not using one. | ✓ |
| Outcomes | VI | Describe all prespecified primary and secondary outcomes, including how and when assessed. | ✓ |
| Outcomes | 9 | Describe how safety and harm outcomes were assessed. Describe which, and how, other XR-specific outcomes were assessed, such as performance, usability, presence, perspectives, and acceptability. | ✓ |
| Sample size | VII | Provide a justification for the sample size. | ✓ |
| Analysis | VIII | Provide a detailed description of how primary and secondary outcomes were analyzed, including any prespecified comparisons or stratifications. |  |
| Protocol alterations | IX | Describe changes to the methods or protocol, including procedures, study outcomes, eligibility criteria, and analysis plan, after study commencement, with reasons, and, if applicable, report whether the study registration was updated. | ✓ |
| Results |  |  |  |
| Participant flow and recruitment | X | Describe the time frame of recruitment and follow-up and the participant flow, including the number of patients screened and included, receiving the intervention, and being included in each analysis. Report if, and why, the study was prematurely terminated. The use of a flow diagram is highly recommended. | ✓ |
| Baseline data | XI | Describe, or add a table depicting, baseline and treatment-related characteristics. If applicable, describe and specify any concurrent measures. | ✓ |
| Main results | XII | Report on all prespecified outcomes that are available. Consider using tables, figures, or graphs to illustrate results. | ✓ |
| XR and human factors | 10 | Include information about the usage of the application, such as duration, frequency, number of sessions, error rates, and number of sessions requiring interruption or discontinuation, including reasons. | ✓ |
| XR and human factors | 11 | If assessed, report on XR-specific outcomes, such as performance, usability, presence, perspectives, and acceptability. | ✓ |
| Safety and harms | 12 | Report on safety and harms, including unintended effects, both during and after using the application. | ✓ |
| Discussion and conclusion |  |  |  |
| Generalizability and impact | 13 | Discuss (potential) impact of study findings and generalizability, including barriers for the use and implementation of the application. | ✓ |
| Safety and harms | 14 | Discuss safety and instances of harm, including their possible effects on study findings, implications for future use of the applications, and whether they can be prevented or mitigated. | ✓ |
| Ethics | 15 | Describe ethical considerations, including benefits and risks, for the current and future use of the application. | ✓ |
| Strengths and limitations | XIII | Discuss study strengths and limitations, including sources of potential bias. | ✓ |
| Conclusion | 16 | Provide a conclusion that accurately interprets study findings, including future perspectives. | ✓ |
| Statements |  |  |  |
| Funding and conflicts of interest | XIV | Disclose any potential conflict of interest, real or apparent, including the funding sources and their roles in the design, conduct, analysis, and report of the study, potential roles of commercial companies, and personal conflicts of interest for each author. | ✓ |
| Application | 17 | Indicate whether the application is a commercial product, it is publicly available, it can be accessed, it complies with the medical device regulations, and whether the application was approved for its intended use by a formal regulatory body or if the study is part of the clinical evaluation for future certification. | ✓ |
